# Effectiveness and safety of *Glycyrrhizae* Decoction for Purging Stomach-Fire in Behcet disease patients: Study protocol for a randomized controlled and double-blinding trail

**DOI:** 10.1097/MD.0000000000010265

**Published:** 2018-03-30

**Authors:** Yong Chen, Dan Luo, Jian-Fei Cai, Chen-Hong Lin, Yan Shen, Jun Zou, Jian-Long Guan

**Affiliations:** Department of Rheumatology and Immunology, Huadong Hospital affiliated with Fudan University, Shanghai, P.R. China.

**Keywords:** Behcet disease, formula, *Glycyrrhizae*, integrative medicine

## Abstract

**Background:**

Behcet disease (BD) is a worldwide-occurred autoimmune disorder and currently lack of optional successful treatment. An ancient traditional Chinese medical formula called *Glycyrrhizae* Decoction for Purging Stomach-Fire (GDPSF) was recorded and nowadays has been observed to be effective for BD patients. However, the strict randomized controlled and double-blinding trail is needed to further assess this alternative medicine.

**Methods:**

To ascertain the potential effects and safety of GDPSF for BD patients and to determine whether combination application of GDPSF and thalidomide could possibly reduce the side effects and increase effectiveness for BD management, we will conduct a randomized, double blind, controlled clinical trial. Patients enrolled will be randomly assigned into 3 groups: GDPSF group, thalidomide group, and integrative group (treated by both GDPSF and thalidomide). Participants will receive treatment for 6 months and accept a 12 months follow-up. Before and after treatment, clinical manifestations, blood tests, thalidomide dosage, remission levels, quality of life, and satisfactory levels will be assessed. The data of assessments on each group before and after treatments will be collected and analyzed through historical control, while between groups through intergroup control. Then statistical analysis will be applied to assess the effects and safety.

**Discussion:**

This study protocol will assess the effects and safety of GDPSF for BD patients GDPSF. Combination application of GDPSF and thalidomide might be a new integrative medical method for BD patients.

**Trial registration:**

Chinese Clinical Registry (ChiCTR-ONC-16009621) on Oct. 2016 http://www.chictr.org.cn/showproj.aspx?proj=16395.

## 1 Background

Behcet disease (BD) is a type of autoimmune disorder that affects multiple parts of the body. The most common symptoms include painful mouth ulcer, genital ulcer, skin lesions, inflammation of parts of the eye, and arthritis. If the activity of the disease was not been well controlled, the inflammatory reaction may progress to the brain or spinal cord, gastrointestinal tract, blood clots, aneurysms, or blindness. Although the events of vital organs involvement is less frequent but can be life threatening.^[1]^

There is no cure available currently. Treatment is aiming at easing the symptoms, reducing inflammation, and controlling the immune system. Drugs may include immunosuppressive medication such as corticosteroids, colchicine, and thalidomide, etc.^[2]^ However, the quality of the evidence for treating the symptoms such as oral ulcers associated with BD is poor.^[3]^

BD was named after Hulusi Behcet (1889–1948), a Turkish dermatologist in 1937. BD is also known as the name “Silk Road Disease” as it is observed with a higher incidence rate among populations living along the historic Silk Road that from Japan and China in the Far East to the Mediterranean Sea, including countries such as Turkey and Iran.^[4]^ Interestingly, a historical Chinese doctor called Zhang Zhongjing (Chinese: Zhang Zhongjing; 150–219) recorded the similar manifestations as Huhuo disease in his work Synopsis of Golden Chamber, dated from more than 1800 years ago. And the syndrome was treated by *Glycyrrhizae* Decoction for Purging Stomach-Fire (GDPSF, Chinese: Gancao Xiexin Tang).^[5]^ GDPSF is investigated to be effective for BD and many other diseases especially gastrointestinal related.^[6,7]^

GDPSF is a formula comprised of multiple herbs, such as, *Glycyrrhiza uralensis* Fisch, *Panax ginseng* CA Mey, *Scutellaria baicalensis* Georgi, and jujube ect. Due to the complexity of the ingredients, few researches investigated the mechanism of its action. Extractive from *Glycyrrhizae*, which plays sovereign role in the decoction, was reported with antiviral activity^[8]^ while BD is regarded to be induced by various pathogenic microorganisms especially herpes simplex virus-1.^[9]^*P ginseng* has favorable impacts to body metabolism and immune function.^[10,11]^ Massive researches in this way may be considering the underline mechanisms of GDPSFs action.

Some clinical studies published in China reported the effect of GDPSF for BD with high remission rate. However, there have been few high quality trials. There has been no trial that combine GDPSF and chemical drugs as an integrative medical method for BD. This study aims to check the effectiveness and safety of GDPSF in BD patients according to strict, high-quality methodology and follow the Consolidated Standards of Reporting Trails (CONSORT) statement for randomized controlled trials of herbal medicine.^[12]^ The results of this trial hopefully to be published in an authorized publication to be accessed to health providers and BD patients worldwide.

## 2 Methods

### 2.1 Objective

The primary objective of this study is to ascertain the potential curative or improvement effects and safety of GDPSF for BD patients with mucocutaneous involvement only. The secondary objective is to determine whether combination application of GDPSF and thalidomide could possibly reduce the side effects caused by thalidomide and increase effectiveness for BD management.

### 2.2 Study design

**Figure 1:**
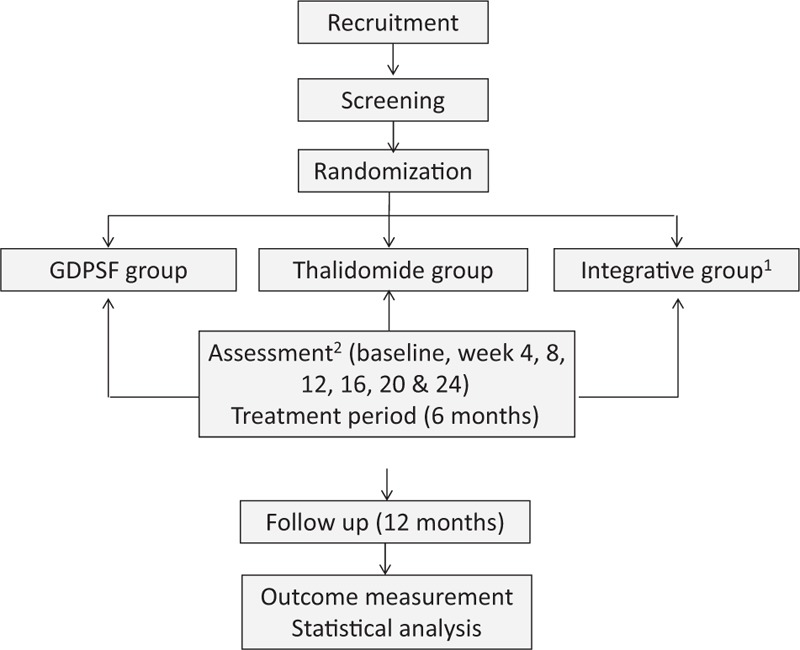
Flow chart of the study. ^1^Integrative group patients receive both thalidomide and Glycyrrhizae Decoction for Purging Stomach-Fire (GDPSF); however, the dosage of thalidomide is range from 25 to 100 mg flexibly according to patients’ treatment sensitivity. ^2^Assessment contents clinical manifestations, blood checkup, and side effects.

This is a single center, double-blinded, randomized, and controlled, parallel-group study. Figure 1 depicts a flow chart of the study. Patients enrolled in this study will randomly be assigned into 3 different groups with respective treatment. Clinical manifestations, blood inflammatory index including erythrocyte sedimentation rate (ESR), C-reactive protein (CRP), and some of the blood biochemical tests will be checked before treatments started (baseline) and will be reexamined on week 4, 8, 12, 18, 20, and 24 during 6 months of treatment to assess the effectiveness and safety. Try to follow up every participant in another 12 months to compare satisfactory rate, complete and partial remission rate, and ineffective rate in different treatment groups.

### 2.3 Study population

This study will be performed in the Rheumatology and Immunology Department of Huadong Hospital affiliated to Fudan University. Patients diagnosed with BD according to the International Criteria for Behcet Disease (ICBD)^[13]^ will be assessed for eligibility. The inclusion and exclusion criteria are presented in Table 1.

**Table 1:**
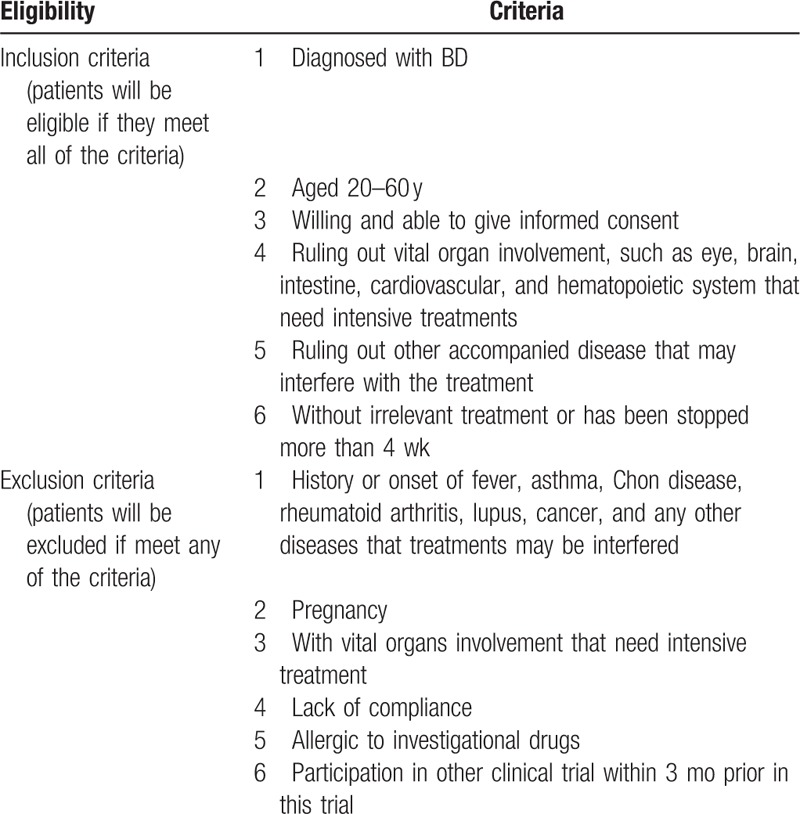
Screening the eligibility of the patients diagnosed with BD.

A patient on admission will receive blood, stool, and urine routine test, ESR, CRP; blood biochemical examination on liver and kidney functions, blood glucose, blood fatty, and electrolytes; coagulation function, autoantibodies, immunoglobulins (Igs), and cancer markers; T-SPORT.TB, hepatitis viruses, HIV, and Treponema pallidum; MRI for skull, X-ray for lung, and ophthalmofundoscope exam; gastrointestinal endoscopy and B-ultrasound for thyroid gland, liver, gallbladder, pancreas, spleen, cardiovascular, urinary, and reproductive systems to screen eligibility of the candidate and record the baseline assessment also.

### 2.4 Ethical issues

The trial will be conducted in accordance with the Declaration of Helsinki and the Ethical Guidelines for Clinical Research, and the trial protocol has been approved by the Research Ethical Committee of Huadong Hospital (No: 2014K050). This trial was registered with ISRCTN at Chinese Clinical Registry (ChiCTR-ONC-16009621) on October 2016. Signed consent form will be obtained from BD patients participated prior to randomization.

### 2.5 Randomization and blinding

To minimize selection bias, all eligible patients will be randomized in a 1:1:1 ratio to the 3 different intervention groups. Patients will be allocated to each group in turn based on the turn of patients’ identification number generated by Hospital Information System (HIS). Randomization will be performed by a professional, independent statistician who is not involved in the recruitment process of this study. Randomization information will be kept secure in Good Clinical Practice (GCP) Center of Huadong Hospital, neither patients nor doctors know the specific group a participant joined in, and will not be opened until after statistical analysis or in the case of a medical emergency.

### 2.6 Groups and interventions

Included patients will be randomly allocated into 3 groups: GDPSF group, thalidomide group, and integrative group. GDPSF group will receive GDPSF and placebo pills of thalidomide size, color, and shape made from cheese. Thalidomide group will take 100 mg thalidomide pill every night and placebo water of GDPSF color and amount. Integrative group will receive both GDPSF and thalidomide, which dosage could be flexible, range from 25 to 100 mg as long as the symptoms could be controlled during the treatment period. All of the patients in these 3 groups will take their medications for 6 months duration.

GDPSF composed of 7 herbs, including 12 g *Glycyrrhiza uralensis* (GanCao), 9 g *Scutellaria baicalensis* (HuangQin), 12 g *Pinellia ternate* (BanXia), 3 g *Coptis chinensis* (HuangLian), 9 g *Zingiber officinale* (GanJiang), 9 g *P ginseng* (RenSheng), and 12 piece of Jujube (HongZhao). The components of formula will be decocted in Herbs Dispensary of our hospital by herbs tisanes machine, into 3 sealed bags of liquid, 200 mL for each bag. Patients can save the herbal decoction in refrigerator and warm it in microwave oven before taken.

## 3 Outcome assessments

### 3.1 Primary outcome measures

As the oral and genital ulceration, skin lesions (erythema nodosum) are most common complaints in BD patients. We select a mucocutaneous activity index (MAI) in BD created by Mumcu et al.^[14]^ In this MAI:

- Presence of the lesion was coded as 1 for actives and 0 for inactives (0 vs 1 point)
- Pain was evaluated by 100 mm-visual analogue scale (VAS; 0: no pain–100: severe pain) by patients. Then, the VAS score was categorized to calculate the score as follows: ≤10: 0; 11–20: 1; 21–40: 2; 41–60: 3; 61–80: 4; 81 and over: 5 points.
- Functional status was evaluated by a 5-point Likert-type scale: none of the time (0 points), little of the time: 1 point, some of the time: 2 points, most of the time: 3 points and all of the time: 4 points. Mean score was used in the index (Table 2).

**Table 2:**
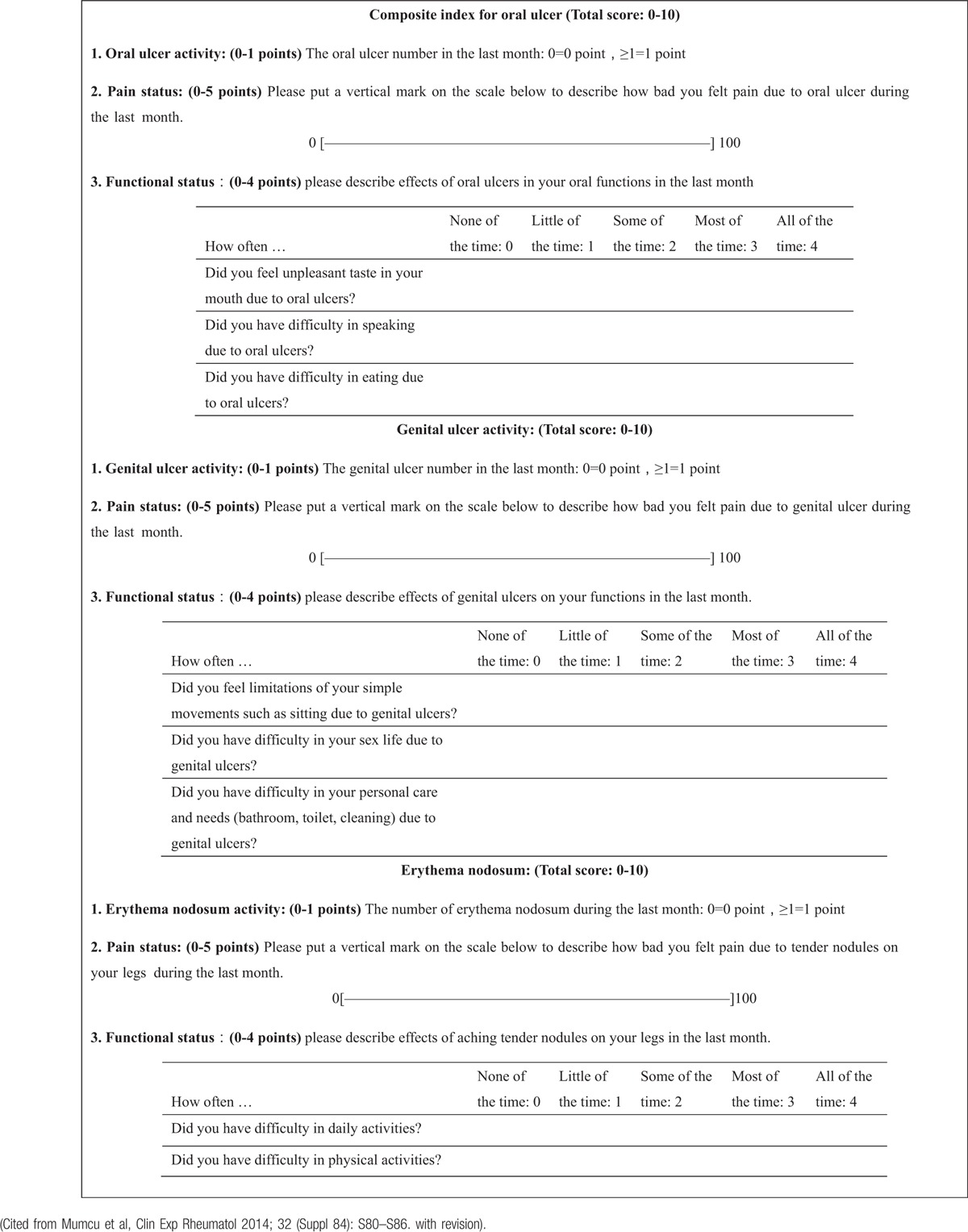
Mucocutaneous activity index and its subscales in BD.

Based on the follow-up of the MAI and other-related symptoms in BD, the remission ratio after treatment will be accessed. The remission degree will be divided into 4 levels:

- Cured: All of the BD manifestations disappeared, rare relapse (less than 2 times a year) after stop treatment for more than 1 year.
- Complete remission: Most of the BD manifestations disappeared (MAI reduce ≥3 points), occasionally relapse (less than 4 times a year), but could easily manage by the treatment.
- Partial remission: Some of the BD manifestations disappeared; the frequency of relapse is less than before treatment.
- No effect: BD manifestations not respond to the treatment, relapse during the treatment.

### 3.2 Secondary outcome measures

Blood inflammatory index including ESR, CRP, and some of the blood biochemical tests such as IgA, IgD, IgE, and IgM, and gamma-glutamyl transpeptidase will be checked before treatments started (baseline) and will be reexamined on week 4, 8, 12, 18, 20, and 24 during 6 months of treatment to assess the effectiveness.

**Figure 2:**
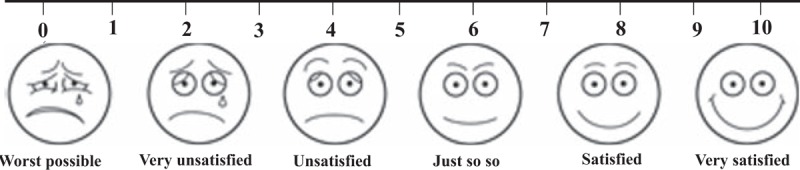
Patients general satisfaction to the treatment.

Satisfactory ratio on effectiveness: Every half a year, the controlling on the BD symptoms will be evaluated by patients’ own intuition. VAS for pain is mimicked by the satisfaction scale (Fig. 2).

Dosage of thalidomide in patients of integrative group will be recorded. This will to evaluate when combination application with GDPSF, if dosage of thalidomide dosage could be decreased.

Quality of life for BD: The quality of life BD, developed by Gill Gilworth et al, provides us with a simple, reliable, and valid tool for assessing the influence of interventions for BD and for evaluating models of service delivery.^[15]^ It is well accepted by patients and has scaling and psychometric properties.

## 4 Safety assessment

Based on current knowledge, GDPSF has rarely side effects, and unlikely cause adverse events (AEs). Anyway, uncomfortable react after taking will be recorded. Potential AEs caused by thalidomide in adults include neutropenia, leukopenia, lymphopenia, anemia, thrombocytopenia, peripheral neuropathy, confusion, depressed mood, reduced coordination, tremor, dizziness, tingling, numbness, constipation, peripheral edema, liver damage, menopause, heart failure, difficulty breathing, interstitial lung disease, lung inflammation, vomiting, dry mouth, rashes, dry skin, fever, weakness, and a sense of unwellness.^[16]^

The contact information of the experimenters will be informed to every subject. If there are any AEs, participants can inform the investigator anytime. Appropriate treatment to the AEs occurred will be provided. In case of serious AEs, the subject shall stop the medicine, and been recommended to other treatment options. All of the AEs in 3 groups will be recorded for assessment.

### 4.1 Statistical considerations

#### 4.1.1 Sample size calculation

One of the aim in the study aims to detect the symptoms easing effect of GDPSF in BD through historical control. The complete remission of symptoms rate reported ranged from 22% to 81%, while effective rate generally above 70%.^[17]^ The reported complete remission rate of thalidomide for BD symptoms ranges from 6% to 81%.^[18,19]^ We also interested in the effects of GDPSF or its combination application with thalidomide compared with thalidomide alone, especially on whether GDPSF improved outcomes or reduce thalidomide intake and its side effects.

Since no strict randomized controlled trials have been previously performed, we proposed this study to evaluate the feasibility of a large-scale clinical trial. Anticipating a 20% dropout rate, a total of 150 patients, with 50 patients in each group, will be recruited for this pilot study.

### 4.2 Statistical analysis

**Figure 3:**
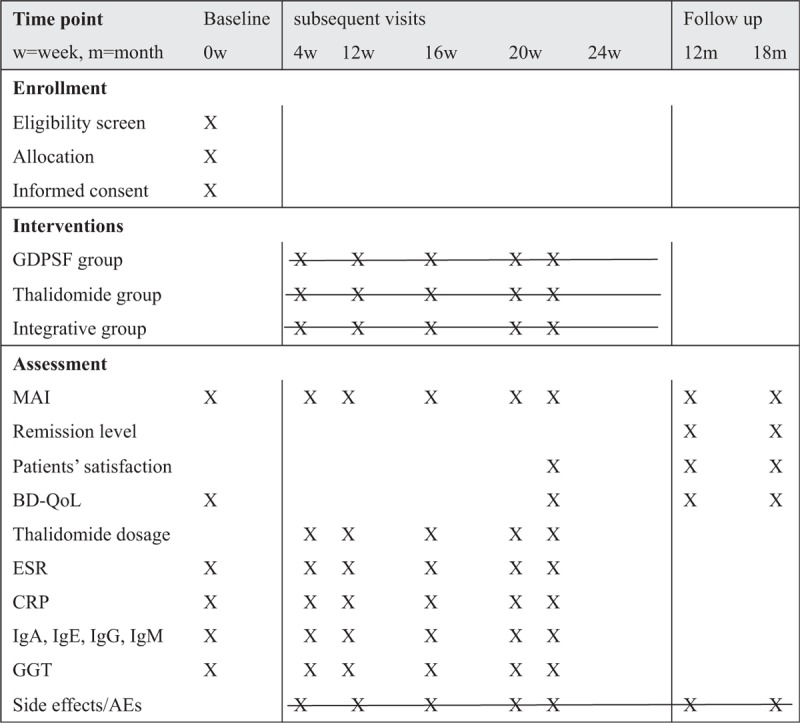
Schedule of enrollment, interventions, and assessments (using the SPIRIT 2013 statement). AE = adverse event, BD-QoL = quality of life for Behcet disease, CRP = C-reactive protein, ESR = erythrocyte sedimentation rate, GDPSF = Glycyrrhizae Decoction for Purging Stomach-Fire, GGT = gamma-glutamyl transpeptidase, Ig = immunoglobulin, MAI = mucocutaneous activity index.

This is an 18 months clinical trial, in which participants will receive treatment for 6 months and accept a 12 months follow-up. During their treatment, participants will visit investigators every 4 weeks 5 times in all. Their clinical manifestations (MAI), blood tests, thalidomide dosage in thalidomide, and integrative groups will be assessed. Participants will receive 2 times of follow-up for 12 months after treatment, their remission levels, quality of life, and satisfactory levels will be assessed. Figure 3 summarizes the study schedule of enrollment, interventions, and assessments according to the SPIRIT 2013 statement. The data of assessments on each intervention group before and after treatments will be collected and analyzed through historical control, while between groups through intergroup control method.

IBM SPSS 21 Statistics will be applied for the analysis. Measurement data that followed normal distribution will be recorded as mean ± SD and analyzed by ANOVA or *t* test, otherwise, Kruskal–Wallis will be applied. Count data will be analyzed by χ^2^ test. The statistical significance level was set at *P* < .05 for all the tests.

## 5 Discussion

In this pilot study, we chose mucocutaneous type of BD patients as out eligible patients, because, if the disease involved vital organs already, intensive treatments will be needed, which makes the simple application of GDPSF or thalidomide not safe for patients. The symptoms caused by the disease especially recurrent oral and genital ulceration cause pain and decrease quality of life in BD patients, lead them suffer physically and psychologically.^[20]^ As a result, it is not proper to give a BD patient placebo treatment as a control. Thalidomide is one of the most common options to erase symptoms in adult BD patients. It provided a useful therapeutic option in severe oral and genital ulceration, which had not responded to other therapies.^[20]^ Therefore, besides of historical control, thalidomide treatment group will set as a control.

Traditional Chinese medicine (TCM) gained afflatus from war that similar principles dominate in both military and medicine, which is to prescribe drugs as monarch, minister, assistant, and guide in a formula according to military action strategies or country governance. In the formula of GDPSF, the *G uralensis* plays the role of monarch, which targets the disease or main symptoms suffered. There has been numerous of in vivo and in vitro researches on antiinflammatory activity from *G uralensis*.^[21,22]^ Extracts from *S baicalensis* also showed activity of antiinflammation, antitumor, and attenuates oxidant stress.^[23,24]^ Researches on *P ternate* shows antidepression, gastrointestinal regulation,^[25,26]^ and effects on blood pressure.^[27]^*C chinensis* shows activity of antimicrobial,^[28]^ while mycobacterial such as *Streptococcus* were though as one of the risks to induce the onset of oral ulcers in BD.^[29]^ The multiple functions of *S baicalensis*, *C chinensis*, and *P ternate* can be used to assistant monarch to enhance the effectiveness for the main disease or symptoms, play a role of minister. Potential side effects in the formula based on TCM would relieve by *P ginseng*, which has been richly investigated. As the chemical elements are extremely complex in n herb formula, and considering the relation between parts and whole, it is impossible to illustrate the GDPSF pharmaceutical actions through current researches on extractives. Good news is the compound formula is gaining attention.^[30]^

There is extensive literature on the chemical constituents and bioactivities of *P ginseng*. The chemical constituents isolated and detected from ginseng show the biological activities of immunoregulatory activity, neuroregulation activity, wound and ulcer healing activity, etc.^[31]^ Thus, we hypothesize, GDPF may reduce the events like leukopenia, peripheral neuropathy caused by thalidomide. Therefore, in this research, we set an integrative group to combine the application of GDPSF and thalidomide, hopefully to enhance the treatment outcomes and reduce side effects.

The Silk Road is an ancient trading route that were for centuries central to cultural interaction originally through regions of Eurasia connecting the East and West and stretching from the Korean peninsula and Japan to the Mediterranean Sea. Underling the guideline of One Belt and One Road policy of Chinese government, we hope TCM can serve more people in the world. Through this ROTs, we hope to provide a new evidence-based treatment option for BD patients.

## Author contributions

**Conceptualization:** Y. Chen.

**Writing – original draft:** Y. Chen.
